# Isolated Abdominal Wall Metastasis of Endometrial Carcinoma

**DOI:** 10.1155/2014/505403

**Published:** 2014-09-30

**Authors:** Rita Luz, Rui Leal, Jorge Simões, Matilde Gonçalves, Isabel Matos

**Affiliations:** Centro Hospitalar de Setúbal, Rua Camilo Castelo Branco, 2910-446 Setúbal, Portugal

## Abstract

A woman in her mid-60s presented with a bulky mass on the anterior abdominal wall. She had a previous incidental diagnosis of endometrial adenocarcinoma FIGO stage IB following a vaginal hysterectomy. Physical exam and imaging revealed a well circumscribed bulging tumour at the umbilical region, measuring 10 × 9 × 9 cm, with overlying intact skin and subcutaneous tissue. Surgical resection was undertaken, and histological examination showed features of endometrial carcinoma. She began chemotherapy and is alive with no signs of recurrent disease one year after surgery. This case brings up to light an atypical location of a solitary metastasis of endometrial carcinoma.

## 1. Introduction

Uterine cancer is the most frequently diagnosed gynaecological malignancy in developed countries. In 2008, more than 288,000 new cases were registered in the world, with a mortality rate of 2.2 per 100,000 women [1].

The majority of women with endometrial carcinoma are diagnosed with localized early stage disease, yielding a high survival rate. The first step of treatment is complete surgical staging that can be followed by adjuvant radiotherapy and chemotherapy or both. In early stage disease, adjuvant radiotherapy has shown to significantly reduce locoregional recurrence, although it does not appear to increase overall survival or distant recurrence rates. Adjuvant chemotherapy can be considered in high-risk patients, but compared to radiotherapy alone, no differences were found in overall survival and progression-free survival, although further studies are needed. Combined modality treatment in high-risk patients was associated with a reduction in risk of recurrence or death in two randomized clinical trials and is the subject of the ongoing PORTEC 3 study [2]. For medically inoperable patients, radiation therapy is useful and can provide some measure of pelvic control and long-term progression-free survival [3].

Following primary treatment, the overall recurrence risk ranges from 8 to 19% [4]. Treatment should be individualized depending on location and performance status. Surgical resection of isolated metastasis might be considered, but chemotherapy is usually the mainstay of treatment. The diagnosis of distant metastases carries an overall poor prognosis, and median survival is reduced to only about one year [2, 5].

The authors describe a case of an atypical location of endometrial carcinoma metastasis on the anterior abdominal wall without other signs of advanced disease.

## 2. Case Presentation

A Caucasian woman in her mid-60s, gravida 1 para 1, with toxic multinodular goiter with an intrathoracic extension, hypertension, chronic anaemia, and obesity (BMI 36 Kg/m^2^) presented 3 years before with stage IV uterine prolapse and occasional vaginal spotting related to ulceration of the protruding cervix. Preoperative evaluation included a pelvic ultrasound that showed an enlarged uterus (L 89 × AP 58 × W 61 mm) with various small fibroids (around 20 mm) that distorted endometrial cavity hindering an accurate measurement of endometrial thickness. Both ovaries were normal in shape and size, and no malignancy was suspected. The optimization of the underlying diseases delayed surgery for several months. In July 2012, a vaginal hysterectomy was performed. Intraoperatively, inspection of both ovaries was unsuspicious. The histopathology examination revealed an enlarged uterus distorted by the presence of multiple fibroids and an intracavitary tumour invading more than half of the myometrium. The tumour was grade 3, poorly differentiated endometrial adenocarcinoma, without vascular space invasion, FIGO stage IB. Given her general condition and absence of signs or symptoms of the disease, despite incomplete surgical staging and high intermediate-risk disease, it was decided by a multidisciplinary team of gynecologic surgeons, pathologists, medical, and radiation oncologists to proceed with adjuvant radiotherapy. Chemotherapy was not considered due to her medical and social background. The patient received 50 Gy (25 fractions of 2 Gy) of external pelvic irradiation and 24 Gy (4 fractions of 6 Gy) of brachytherapy to the vaginal cuff.

Around six months after surgery, she noted a painless nodule on the anterior abdominal wall. On physical examination, there was a large mass on the left side of the umbilical region, measuring 10 × 9 cm. The mass was hard on palpation, with irregular edges and surface, and was well circumscribed but fixed to surrounding soft tissue. Overlying skin and umbilicus had a typical appearance. General examination including the pelvic exam was otherwise unremarkable.

**Figure 1:**
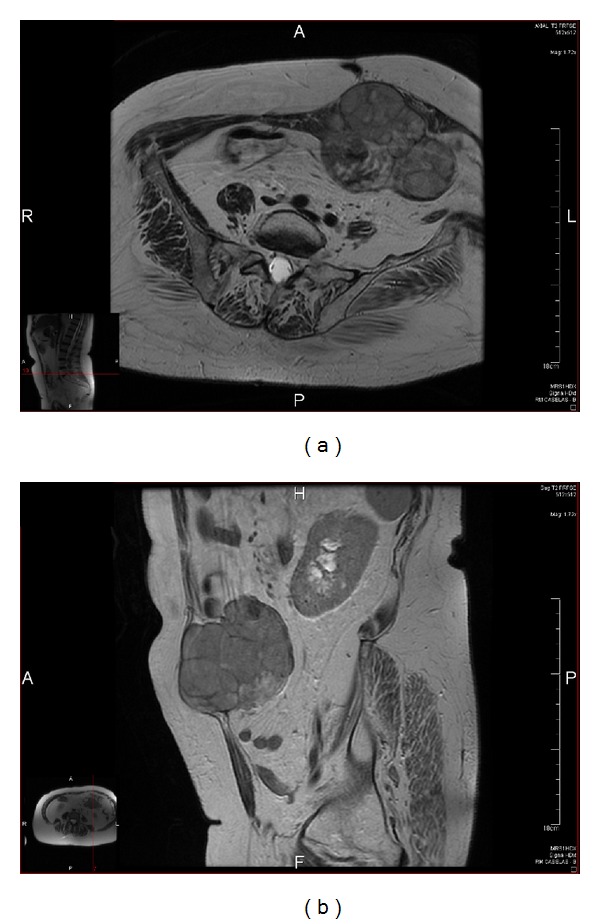
MRI demonstrates the tumour on the left side of the anterior abdominal wall protruding into the abdominal cavity. (a) Axial T2-weighted image and (b) sagittal T2-weighted image.

Laboratory investigations were normal except for hypochromic microcytic anaemia (Hb 9.6 g/dL), lactate dehydrogenase mildly raised (333 UI/L), and elevated CA 125 (478 UI/mL). Magnetic resonance imaging (MRI) (Figures 1(a) and 1(b)) revealed a tumour on the umbilical region, slightly shifted to the left side, protruding into the abdominal cavity, measuring 10 × 9 × 9 cm. The tumour was well demarcated from the surrounding fat tissue and muscular layer with an anterior relationship with the umbilicus. It was highly heterogeneous with central areas of necrosis. There was no local or regional recurrence, pelvic lymph node involvement, or hepatic metastases. Thoracic computed tomography (CT) showed no pulmonary metastases.

**Figure 2:**
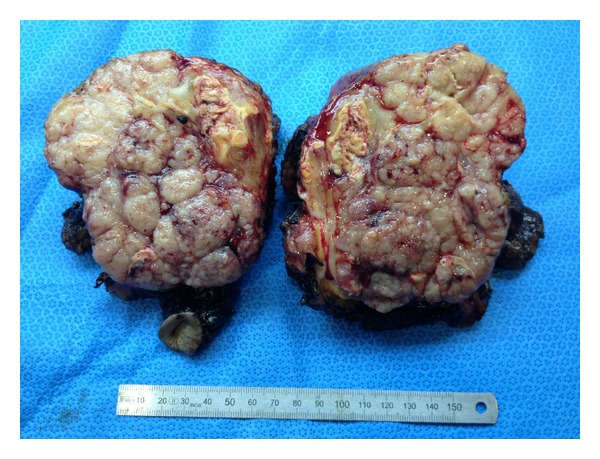
Cross section of the solid tumour with central necrosis and intact umbilicus.

**Figure 3:**
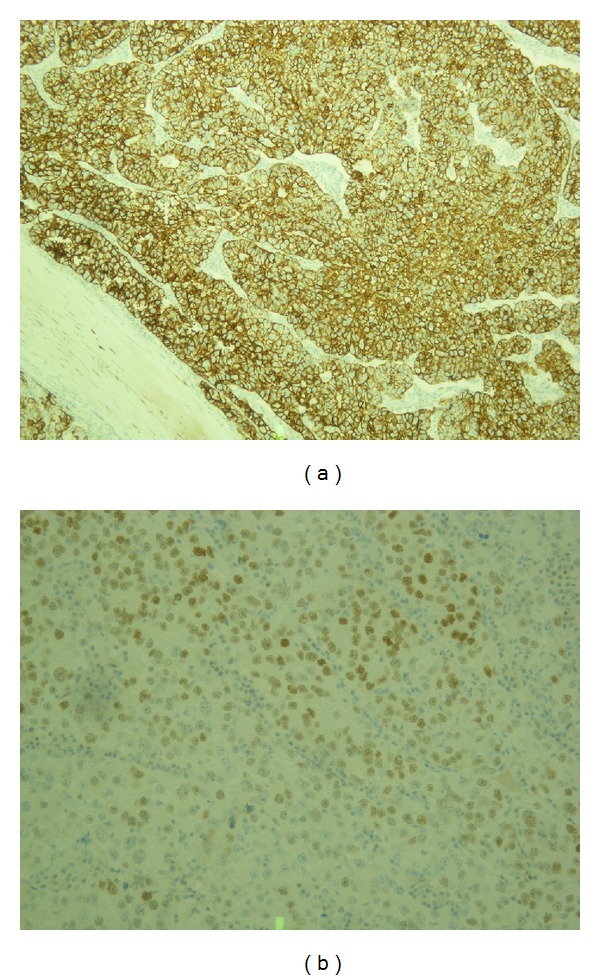
Microphotograph showing a section of the abdominal wall mass with cytokeratin 7 (a) and oestrogen receptor (b) positive immunohistochemistry.

Surgical resection of the tumour was performed followed by peritoneal washing and bilateral salpingo-oophorectomy. Reconstruction of the abdominal wall was accomplished with the use of a synthetic mesh. The cross section of the tumour is shown in Figure 2. Histological examination revealed a solid tumour lined by adipose tissue and peritoneum with the same histology as the primary endometrial carcinoma. Immunohistochemistry showed positivity for cytokeratin (CK) 7 and oestrogen receptor and was negative for CK20 (Figures 3(a) and 3(b)). The peritoneal washing was positive for neoplastic cells, and both fallopian tubes and ovaries were not invaded.

She began chemotherapy one month after surgery with paclitaxel (175 mg/m^2^) and carboplatin (300 mg/m^2^) and completed six cycles. At the end of treatment, radiologic imaging with thoracic and abdominal CT and pelvic MRI did not show any signs of recurrence. More than one year after surgery, the patient is alive and asymptomatic.

## 3. Discussion

Endometrial carcinoma can spread through several routes, depending on the histological type and local invasion. The patterns of spread include direct extension, lymphatic and haematogenous dissemination, and retrograde passage of neoplastic cells through the fallopian tubes [6]. The anterior abdominal wall, especially the umbilical region, has a rich arterial supply, an anastomotic venous network, and a lymphatic system that drains cranially and caudally to several lymphatic chains including pelvic and para-aortic lymph nodes. All these systems could be involved in the dissemination of neoplastic cells to the soft tissue of the abdominal wall. Another explanation may be related to peritoneal direct extension and spread through embryonic remnants [7, 8].

Endometrial cancer relapses are most frequently localized in the vaginal cuff, pelvic and para-aortic lymph nodes, peritoneum, lungs, and liver. Unusual sites include abdominal wall and muscle (2–6%), spleen (1%), central nervous system (<1%), extra-abdominal lymph nodes (0,4–1%), and, more rarely, adrenals, pancreas, and appendix [9–13].

In this case and taking into account its location near the umbilicus, the hypothesis of an umbilical metastasis, also termed Sister Mary Joseph's nodule, was raised. Sister Mary Joseph's nodule most typically presents as an irregular lump on the umbilicus, ranging in size from 0.5 to 2 cm, although there are reports with nodules reaching up to 10 cm. It may be ulcerated and necrotic and have a bloody, mucinous, serous, or purulent discharge. It can be detected before or during diagnosis of the primary tumour or after treatment. This umbilical metastasis is found in 1–3% of patients with gastrointestinal or genitourinary malignancy, including endometrial cancer [7, 14]. There are little more than 30 cases reported in the literature originating from endometrial cancer. The presence of this nodule generally indicates advanced cancer with widespread metastases and, therefore, poor prognosis [15–18]. In patients with good clinical state, a combination of surgery and adjuvant therapy can improve survival, but in some cases only palliative care is feasible [7]. In this case, although anatomically close, the abdominal wall mass seems separated from the umbilicus, does not display the typical signs of Sister Mary Joseph's nodule, and was not associated with widespread disease.

Abdominal wall metastases have also been linked to surgical incision, regardless of the surgical approach (laparotomy or laparoscopy). Although rare, it has been described in cases of endometrial carcinoma [19–23]. The exact mechanism of this event is usually explained by haematogenous dissemination to the site of recent trauma, seeding of neoplastic cells after direct contact between the tumour and the wound, effects of pneumoperitoneum, surgical technique, and local immune response. Management of port-site metastases and laparotomy wound recurrences includes an extensive workup to rule out other metastases. In the absence of distant disease, enlarged excision and exploratory laparotomy or laparoscopy should be attained [24]. In this case, the first surgery was vaginal, and the patient had no history of abdominal surgery, which discards this hypothesis.

Park and Hwang reported a case of a postmenopausal woman with an abdominal wall metastasis in stage IA serous endometrial adenocarcinoma, eight months after surgical staging. Treatment consisted of surgical excision and chemotherapy, and three years after surgery she is alive with no signs of disease [25]. Comparing to the current case site and time to recurrence were similar as well as the treatment chosen. Most guidelines only support surgical resection in selected patients with good performance status. Pelvic exenteration or even partial vaginectomy may be considered in pelvic central recurrences, especially after radiotherapy failure. Other pelvic, abdominal, retroperitoneal, and extra-abdominal recurrences can be amenable to salvage cytoreductive surgery [2, 3]. In small retrospective studies, optimal debulking significantly improved survival and patients who had solitary metastasis were more likely to achieve complete cytoreduction [26–28]. Campagnutta et al. reported a 30% rate of major surgical complications, which supports the importance of proper selection criteria [26]. Resection of abdominal wall tumours with negative margins is feasible but often requires reconstruction of the abdominal wall defect with prosthetic mesh [29]. Chemotherapy can also be considered, especially in unresectable or disseminated metastases. Combination regimens with paclitaxel and carboplatin or cisplatin are frequently used for recurrent endometrial cancer, based on a good response rate on ovarian cancer studies [3].

This case highlights an unusual location of a solitary metastasis of endometrial cancer on the soft tissue of the abdominal wall. Almost all cases reported in the literature are diagnosed after primary treatment and are related to the surgical incision. In this case, it is difficult to know exactly how neoplastic cells implanted in the soft tissue and developed into a mass. Surgical resection was feasible and the patient completed chemotherapy with no signs of recurrence after one year.
